# Metabolome analysis of genus *Forsythia* related constituents in *Forsythia suspensa* leaves and fruits using UPLC-ESI-QQQ-MS/MS technique

**DOI:** 10.1371/journal.pone.0269915

**Published:** 2022-06-28

**Authors:** Lingdi Liu, Yu Sun, Chunxiu Wen, Tao Jiang, Wei Tian, Xiaoliang Xie, Xusheng Cui, Ruike Lu, Jiaxing Feng, Aihong Jin, Saiqun Wen, Wei Wei

**Affiliations:** 1 Institute of Cash Crops, Hebei Academy of Agricultural and Forestry Sciences, Shijiazhuang, Hebei, China; 2 Hebei Medicinal Plant Engineering Technology Research Center, Shijiazhuang, Hebei, China; 3 Shijiazhuang Institute of Pomology, Hebei Academy of Agricultural and Forestry Sciences, Shijiazhuang, Hebei, China; 4 Shijiazhuang Yiling Pharmaceutical Co, Ltd, Shijiazhuang, Hebei, China; Institute for Biological Research, University of Belgrade, SERBIA

## Abstract

*Forsythia suspensa* is a traditional Chinese herb. Its numerous metabolites have important roles, as they possessed a wide range of biological activities. This study explored the accumulations of *F*. *suspensa* metabolites by performing widely targeted metabolomic analysis. The metabolites were studied at four stages of fruit development. Metabolites in the fruits and leaves of *F*. *suspensa* during fruit development included phenolic acids, flavonoids, lipids, lignans and coumarins, amino acids and their derivatives, terpenes, organic acids, nucleotides and their derivatives, alkaloids, quinones, steroids, and tannins. Fourteen *Forsythia* related metabolites were detected. Their contents varied among the developmental stages. Statistically significant correlations were found between the levels of forsythoside B and 11-methyl-forsythide, and forsythialan B and phillygenin, in both leaves and fruits. According to the correlation analysis between metabolites, *Forsythia* related metabolites were divided into two classes and five subclasses. In total, 33 compounds presented significant correlations in both fruits and leaves, which indicated the potential relationship in the synthesis of *Forsythia* related metabolites. Forsythialan B and phillygenin were both negatively correlated with L-valine, while Z-6,7-epoxyligustilid was positively correlated with both compounds. The quality control compounds forsythiaside A and phillyrin were positively and negatively correlated with uracil, respectively. These metabolomics results may facilitate the biosynthesis of *Forsythia* related metabolites.

## Introduction

*Forsythia suspensa* (Tunb.) Vahl is widely used in medical applications [1–4]. Previous studies indicated that the compounds present in *F*. *suspensa* include phenylethanoid glycosides, triterpenoids, lignans, and flavonoids. All have pharmacological activities [2]. The medical effects of some of the *Forsythia* related compounds have been tested in vivo and in vitro. The results indicate that *F*. *suspensa* can serve as an effective antipyretice, detoxicant, and anti-inflammatory agent for the treatment of various infectious diseases [2–6]. An overview of the pharmacology of *Forsythiae Fructus* (the dry fruits of *Forsythia suspensa*) reported its anti-inflammatory, antibacterial, antiviral, antioxidant, antitumor, antidiabetic, antihyperlipidemic, antiandrogenic alopecia, antivomiting, antiaging and anti-obesity activities, as well as neuroprotective, hepatoprotective and vasorelaxant effects [7]. Most studies on *F*. *suspensa* have investigated the medicinal values of its active compounds and their use for drug development, and some studies have attempted to identify novel compounds in *F*. *suspensa*. However, the biosynthesis and metabolism of the *Forsythia* related metabolites in *F*. *suspensa* are still not fully understood.

The medicinal values vary in different plant organs of the same species due to the different metabolic processes and accumulation of metabolites in various plant tissues, and it is widely believed that the medicinal compounds in *F*. *suspensa* are present mainly in the fruit. However, previous studies have indicated that the medicinal compound, forsythiaside A, exists in leaves, fruits, and even stems, Thus, the leaves of *F*. *suspensa* may have potential medicinal value. Moreover, forsythiaside A has been reported to be even more abundant in leaves than in fruits. Differences in the levels of the chemical components of *F*. *suspensa* among plant organs have been confirmed. It also appears that all *F*. *suspensa* tissues likely contain many other medicinal compounds, although differences in abundance of these compounds among plant organs are also likely [8]. Besides, the amounts of the metabolites are varying depending on the stage of development, which greatly effects the medicinal metabolites production [9].

Studying the chemical composition and contents of the active compounds of herbal plants is complicated, as many species-distinctive metabolites are derived from secondary metabolic processes [10]. Secondary metabolites have important regulatory roles in resistance to abiotic and biotic stresses and adaptation to the environment. Thus, unique metabolites of different species may be formed during adaptation to growing conditions. Previous studies have isolated and identified various medically active compounds in the fruits of *Forsythia suspensa*, including the structures of rengyosides A, B and C [11]. Numerous fruit-derived metabolites have been isolated from previous studies. Phillyrin, which exists in family Oleaceae, was identified as the main component of *F*. *suspensa* [12]. Forsythiaside and forsythiaside A were isolated from air-dried fruits of *F*. *suspensa*. Previous studies demonstrated their anti-inflammatory and antioxidant effects [13, 14]. Knowledge of metabolites in herbal plants is very important. Metabolome studies [15–21] have shown positive roles in identifying the functions of multiple compounds in plants, including abiotic and biotic stress and medicinal aspects. With the wide applications of metabolomes in plant science studies, various mechanisms of environment adaptation and target compound biosynthesis in plants have been identified. In addition, the biosynthesis and metabolism of a large range of medicinal compounds have been elucidated [19].

Previous studies on *F*. *suspensa* have investigated medicinal compounds in different plant organs in terms of their effects on diseases and their metabolism in vivo [19–21]. The biosynthesis and metabolic pathways of *Forsythia* related metabolites have received little attention and remain poorly understood [20–22]. Although, the use of enzyme assays has revealed the biosynthesis of lignans and iridoids in *F*. *suspensa* compounds [23, 24].

In this study, we conducted a targeted metabolomic analysis of metabolites in the leaves and fruits of *F*. *suspensa* at four fruit developing stages to investigate the vast *Forsythia* related metabolites simultaneously to analyze their potential associations and the correlations with other metabolites.to provide basic theoretical supports on their biosynthesis studies. We also analyzed the dynamics of *Forsythia* related medicinal metabolites in fruits and leaves to determine the optimum harvesting time and plant organ.

## Materials and methods

### Plant material

The *Forsythia suspensa* plants were located in Jingxing, Shijiazhuang, Hebei, China. The leaves and fruits were sampled at four fruit developing stages in 2019. About twenty uniform asexual propagation seedlings of *Forsythia suspensa* were used for this study. We sampled fifty fruits which have relatively uniform growth and physiological state each sampling time as one biological replicate, and the fourth leaves were sampled with the same method. Each sampling was repeated three times to reduce the error and ensure measurement data validation and accuracy. The sample were collected around ten a.m. each time. After sampling, the samples were transported to the laboratory and cleaned in distilled water, and then put into liquid nitrogen and stored at −80°C. We sampled the fruits and leaves every 50 days since May 30th. The sampling dates in 2019 were as follows, May 30th, July 20th, September 10th and October 30th. The four stages were as follows: early-stage (T1), mid-stage (T2), mid-late-stage (T3) and late-stage (T4).

### Sample preparation and extraction

The leaf and fruit samples were freeze-dried to extract the metabolites in the tissues. The freeze-dried samples were crushed using a mixer mill (MM 400, Retsch) with a zirconia bead for 1.5 min at 30 Hz. We weighted 100 mg powder of each sample and extracted overnight at 4°C with 0.6 ml 70% aqueous methanol (HPLC grade, Merck, www.merckgroup.com/). Following centrifugation at 10, 000 g for 10 min, the extracts were absorbed (CNWBOND Carbon-GCB SPE Cartridge, 250 mg, 3 ml; ANPEL, Shanghai, China, www.anpel.com.cn/cnw) and filtrated (SCAA-104, 0.22 μm pore size; ANPEL, Shanghai, China, http://www.anpel.com.cn/) before ultra-performance liquid chromatography-tandem mass spectrometry (UPLC-MS/MS) analysis.

### UPLC and electrospray ionization triple quadrupole linear ion trap (ESI-Q TRAP-MS/MS) conditions

The sample extracts were analyzed using an UPLC-ESI-MS/MS system (UPLC, Shim-pack UFLC SHIMADZU CBM30A system, www.shimadzu.com.cn/; MS, Applied Biosystems 4500 Q TRAP, www.appliedbiosystems.com.cn/). The analytical conditions were as follows, UPLC: column, Agilent SB-C18 (1.8 μm, 2.1 mm 100 mm); The mobile phase was consisted of solvent A, pure water with 0.1% formic acid (HPLC grade, Merck, www.merckgroup.com/), and solvent B, acetonitrile (HPLC grade, Merck, www.merckgroup.com/). Sample measurements were performed with a gradient program that employed the starting conditions of 95% A, 5% B. Within 9min, a linear gradient to 5% A, 95% B was programmed, and a composition of 5% A, 95% B was kept for 1min. Subsequently, a composition of 95% A,5.0% B was adjusted within 1.10 min and kept for 2.9 min. The column oven was set to 40°C; The injection volume was 4μl. The effluent was alternatively connected to an ESI-triple quadrupole-linear ion trap (QTRAP)-MS.

LIT and triple quadrupole (QQQ) scans were acquired on a triple quadrupole-linear ion trap mass spectrometer (Q TRAP), API 4500 Q TRAP UPLC/MS/MS System, equipped with an ESI Turbo Ion-Spray interface, operating in positive and negative ion mode and controlled by Analyst 1.6.3 software (AB Sciex). The ESI source operation parameters were as follows: ion source, turbo spray; source temperature 550°C; ion spray voltage (IS) 5500 V (positive ion mode)/-4500 V (negative ion mode); ion source gas I (GSI), gas II(GSII), curtain gas (CUR) were set at 50, 60, and 30.0 psi, respectively; the collision activated dissociation (CAD) was set at high level. Instrument tuning and mass calibration were performed with 10 and 100 μmol/L polypropylene glycol solutions in QQQ and LIT modes, respectively. QQQ scans were acquired as MRM experiments with collision gas (nitrogen) set to 5 psi. DP and CE for individual MRM transitions was done with further DP and CE optimization. A distincitve set of MRM transitions were monitored for each period according to the metabolites eluted within this period.

### Correlation analysis of metabolites in leaves and fruits

The correlation analysis was performed with *Forsythia* related metabolites and other metabolites detected in this experiment from four fruit developing stages. The correlation values between *Forsythia* related metabolites and other metabolites were based on data of their ion intensities. All data were analyzed by bivariate analysis followed by Pearson’s correlation and Two-tailed test of significance among different groups.

Experimental data of *Forsythia* related metabolites contents were analyzed by one-way analysis of variance followed by Tukey’s multiple range test to detect differences among the groups. A *p*-value < 0.05 was considered significant. All statistical analyses were performed using IBM SPSS Statistics 22 software (IBM Corp., Armonk, NY, United States).

## Results

### Analysis of levels of *Forsythia* related metabolites in leaves and fruits

In this study, ultra-performance liquid chromatography tandem mass spectrometry (UPLC-MS/MS) was used to determine the fold-change values of *Forsythia* related metabolites in leaves and fruits. Quantifications utilized a QqQ mass analyzer. The total ion current (TIC) maps of the mixed sample (quality control sample, QC) were used to verify the reliability and repeatability of the experiment (S1 and S2 Figs). The peak shapes of the TIC maps were consistent, indicating that the results of the experimental results were reliable and repeatable. The extracted ion chromatograms (XIC) exhibited the chromatographic peaks corresponding to the mass metabolites (S3 and S4 Figs). The representative TICs an XICs of fruits and leaves at each harvesting time are shown in S5–S36 Figs. The mass spectrum information of *Forsythia* related constituents is shown in S1 Table.

**Fig 1:**
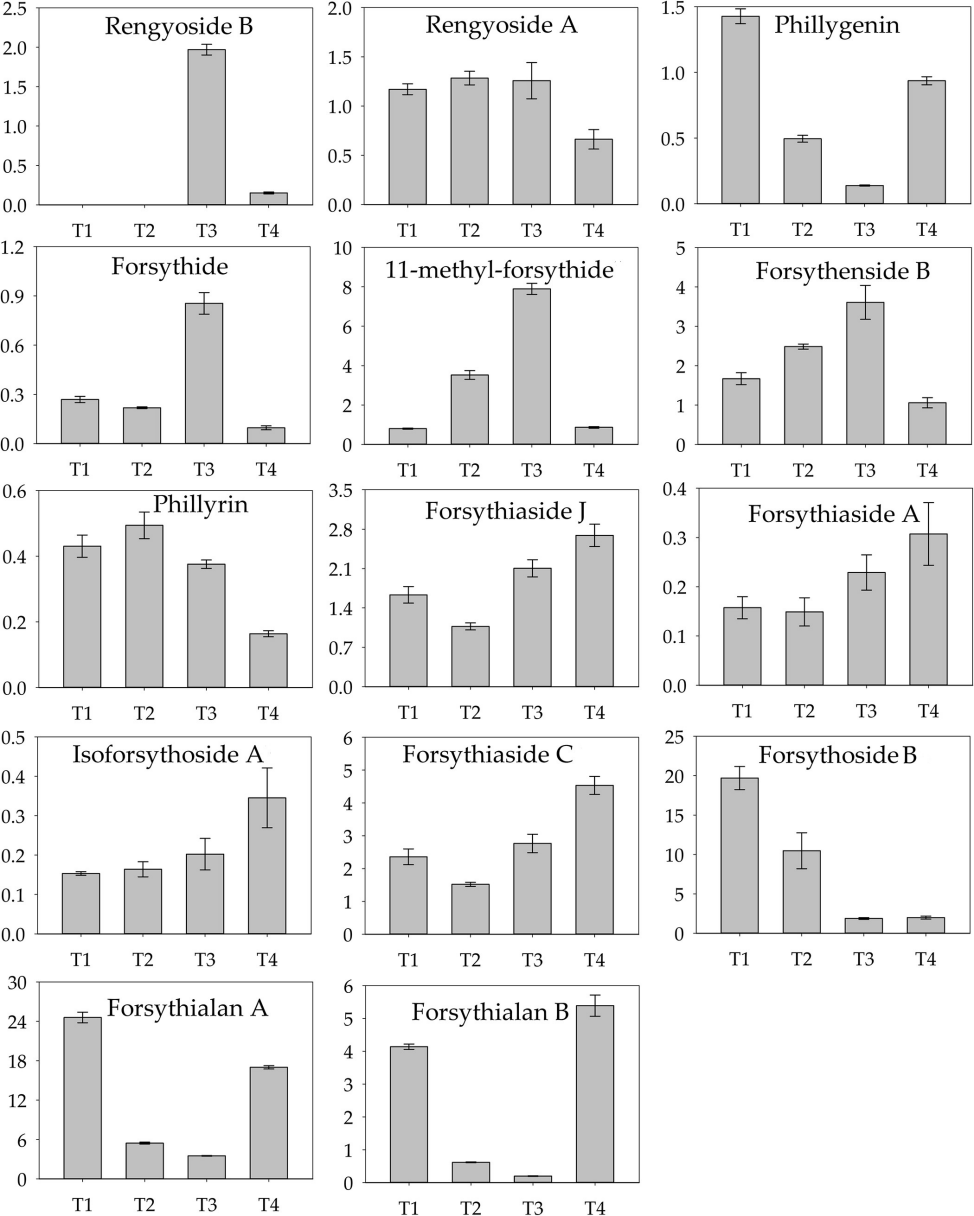
Fold-changes of *Forsythia* related metabolites in leaves and fruits in four harvesting stages.

Forsythiaside C, forsythoside B, rengyoside B, rengyoside, forsythiaside B, forsythiaside J, forsythiaside A, isoforsythoside A, forsythialan B, forsythialan A, phillygenin, phillyrin, 11-methyl-forsythide and forsythide were all detected in fruits and leaves. The *Forsythia* related metabolites were classified as phenolic acids, lignans, and terpenoids, respectively. The fold-change values in the relative levels of metabolites in the fruits and leaves at different fruit developing stages varied markedly (Fig 1). The relative contents of forsythide, phillyrin, forsythiaside A, and isoforsythoside A were higher in leaves than in fruits, with the fold-change values < 1 at the four stages, which indicated that these constituents may be rich in leaves. In contrast, 11-methyl-forsythide, forsythenside B, forsythiaside J, forsythiaside C, forsythoside B, forsythialan A and forsythialan B might be the compounds rich in fruit. Forsythiaside J, forsythiashide A and forsythiaside C represented similar variation among the four fruit developing stages. Forsythiaside A in fruit exhibited lower levels than that in leaves, which differed from forsythiaside J and forsythiaside C. Phillygenin, forsythialan A and forsythialan B showed similar variations in fold-change values. However, phillygenin in fruits did not show much higher levels than that in leaves duing the fruit development process.

### Correlation analysis of *Forsythia* related metabolites

Fourteen metabolites were detected in the fruits and leaves of *Forsythia*. Correlation analyses revealed that forsythiaside C was positively correlated with forsythiaside J, and forsythoside B was positively correlated with 11-methyl-forsythide (Tables 1 and 2). Phillygenin was negatively correlated with rengyoside B, but was positively correlated with forsythialan A. In leaves, forsythiaside A, isoforsythoside A, and rengyoside A were not significantly correlated with any other *Forsythia* related metabolites.

**Table 1:** Correlation analysis of *Forsythia* related metabolites in leaves of *Forsythia suspense*.

Forsythiaside C	Forsythoside B	Rengyoside B	Rengyoside A	Forsythensie B	Forsythiaside A	Forsythensie B	Forsythiaside J	Forsythiaside A	Isoforsythiaside A	Forsythialan B	Forsythialan A	Phillyrin	11-Methy-forsythide	Forsy-thide
**Forsythiaside C**	1													
**Forsythoside B**	-0.775	1												
**Rengyoside B**	-0.78	0.214	1											
**Rengyoside A**	-0.872	0.914	0.420	1										
**Forsythensie B**	0.645	-0.852	-0.114	-0.936	1									
**Forsythiaside J**	.952*	-0.553	-0.915	-0.75	0.502	1								
**Forsythiaside A**	-0.693	0.149	0.891	0.489	-0.3	-0.876	1							
**Isoforsythiaside A**	0.283	0.379	-0.795	0.064	-0.267	0.561	-0.836	1						
**Forsythialan B**	0.853	-0.487	-0.874	-0.501	0.164	0.849	-0.595	0.437	1					
**Forsythialan A**	0.332	0.26	-0.806	0.166	-0.487	0.51	-0.559	0.8	0.716	1				
**Phillygenin**	0.85	-0.375	-.964*	-0.485	0.153	0.913	-0.761	0.624	.971*	0.779	1			
**Phillyrin**	0.935	-0.621	-0.806	-0.851	0.675	.969*	-0.868	0.468	0.704	0.299	0.782	1		
**11-Methyrsythide**	-0.577	.961*	-0.047	0.785	-0.784	-0.304	-0.13	0.618	-0.303	0.437	-0.148	-0.38	1	
**Forsythide**	-0.279	-0.319	0.705	0.064	0.056	-0.557	0.884	-0.949	-0.272	-0.576	-0.492	-0.543	-0.564	1

Note:

*p < 0.05 was considered to demonstrate statistically significant differences.

**p < 0.01 was considered to demonstrate statistically significant differences, the same below.

**Table 2:** Correlation analysis of *Forsythia* related metabolites in fruits of *Forsythia*.

	Forsythiaside C	Forsythoside B	Rengyoside B	Rengyoside A	Forsythensie B	Forsythiaside J	Forsythiaside A	Isoforsythiaside A	Forsythialan B	Forsythialan A	Phillygenin	Phillyrin	11-Methyforsythide	Forsythide
**Forsythiaside C**	1													
**Forsythoside B**	-0.6	1												
**Rengyoside B**	-0.6	0.9	1											
**Rengyoside A**	-0.3	0.88	0.597	1										
**Forsythensie B**	0.78	-0.1	-0.451	0.31	1									
**Forsythiaside J**	0.66	0.18	0.175	0.26	0.559	1								
**Forsythiaside A**	-0.7	0.12	0.484	-0.3	-0.99*	-0.422	1							
**Isoforsythiaside A**	-0.8	0.19	0.526	-0.3	-0.99*	-0.455	0.996*	1						
**Forsythialan B**	0.99*	-0.6	-0.641	-0.4	0.68	0.627	-0.586	-0.653	1					
**Forsythialan A**	0.96*	-0.7	-0.622	-0.5	0.562	0.602	-0.458	-0.532	0.989*	1				
**Phillygenin**	0.99*	-0.6	-0.724	-0.3	0.806	0.55	-0.744	-0.798	0.972*	0.93	i			
**Phillyrin**	0.75	-0.2	-0.5	0.3	0.996*	0.478	-0.996*	-0.999	0.651	0.529	0.793	i		
**11-Methy-rsythide**	-0.4	0.98*	0.919	0.85	-0.08	0.339	0.104	0.157	-0.508	-0.547	-0.521	-0.129	1	
**Forsythide**	-0.3	0.96*	0.769	0.97*	0.163	0.35	-0.166	-0.105	-0.423	-0.503	-0.373	0.13	0.957*	1

Correlations were evident among metabolites in fruits. Forsythiaside C had significant positive correlations with forsythialan B, forsythialan, and phillygenin. Forsythoside B was positively correlated with 11-methyl-forsythide and forsythide. Forsythenside B was negatively correlated with both forsythiaside and isoforsythoside A, and positively correlated with phillyrin. Forsythiaside A was positively correlated with isoforsythoside A and negatively correlated with forsythenside B and phillyrin. Forsythialan A was positively correlated with forsythiaside C and forsythialan B; forsythoside B, 11-methyl-forsythide, and forsythialan B were all positively correlated with phillygenin in both leaves and fruits. However, forsythiaside J and rengyoside B were not significantly correlated with the other *Forsythia* related metabolites.

Despite the inconsistent correlations between *Forsythia* related metabolites in fruit and leaves. Forsythialan B, phillygenin and 11-methyl-forsythide and forsythoside B showed significant and corresponding positive correlations.

### Correlations between *Forsythia* related metabolites and other metabolites

To reduce errors caused by inconsistent amounts of metabolites in fruits and leaves, among the *Forsythia*-related metabolites, we screened for metabolites that exhibited similar correlations in both fruits and leaves. Of the 489 metabolites identified, 225 were selected and classified into 10 types (S2–S4 Tables), comprising phenolic acids (n = 47), lignans and coumarins (n = 21), terpenoids (n = 19), amino acids and their derivatives (n = 11), quinones (n = 2), flavonoids (n = 41), alkaloids (n = 6), nucleotides and their derivatives (n = 13), tannins (n = 1), lipids(n = 18), organic acids (n = 14) and others (n = 31).

**Fig 2:**
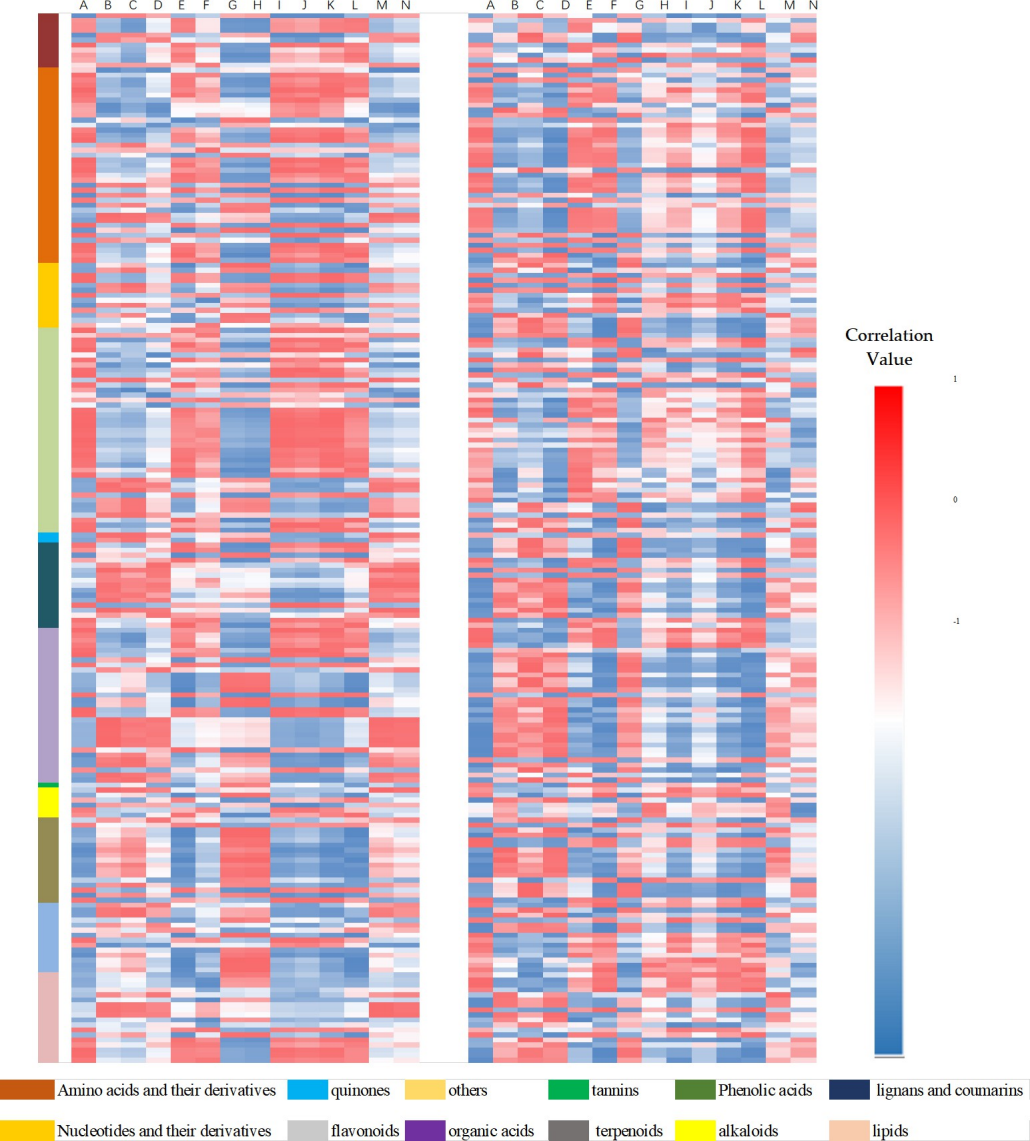
Correlation heatmap of the *Forsythia* related metabolites in leaves and fruits in each harvesting period. The two heatmaps represent the correlations between the metabolites and the *Forsythia* related metabolites in fruits and leaves, respectively. Heatmap of fruits was listed on the left and that of leaves was listed on the right. The left column of the heatmap shows the classification of different types of metabolites. Each *Forsythia* related metabolite is represented by a single column, as follows: A: forsythiaside C, B: forsythoside B, C: rengyoside B, D: rengyoside A. E: forsythiaside B, F: forsythiaside J, G: forsythiaside A, H: isoforsythoside A, I: forsythialan B, J: forsythialan A, K: phillygenin, L: phillyrin, M: 11-methyl-forsythide, and N: forsythide. Red and blue color represent positive and negative correlations.

**Fig 3:**
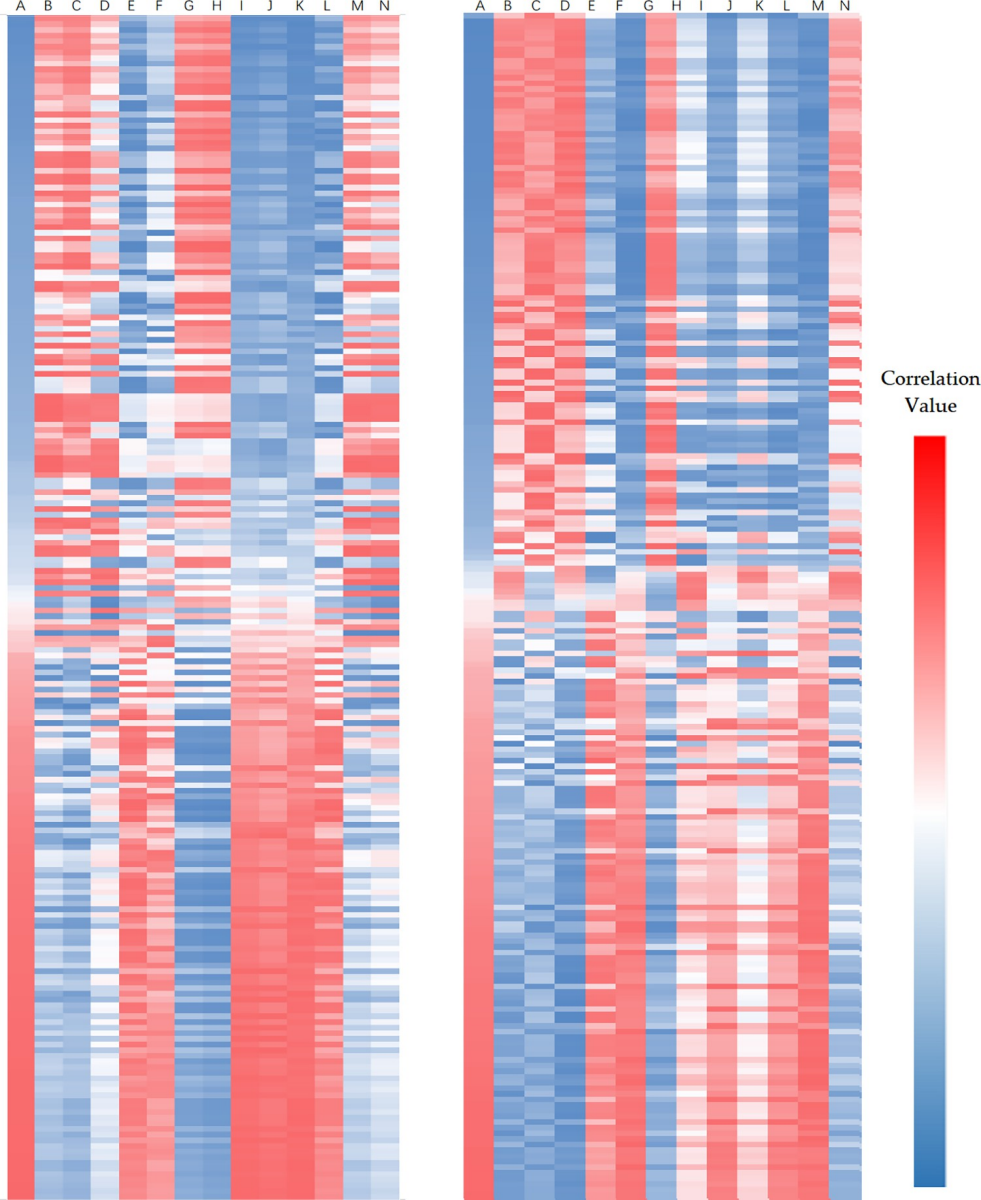
Correlation heatmap of the *Forsythia* related metabolites in leaves and fruits in each harvesting period ranking by correlation values of forsythiaside. The two heatmaps represent the correlations between the metabolites and the *Forsythia* related metabolites in fruits and leaves, respectively. Heatmap of fruits was listed on the left, that of leaves was listed on the right. Each *Forsythia* related metabolites is represented by a single column, as follows: A: forsythiaside C, B: forsythoside B, C: rengyoside B, D: rengyoside A. E: forsythiaside B, F: forsythiaside J, G: forsythiaside A, H: isoforsythoside A, I: forsythialan B, J: forsythialan A, K: phillygenin, L: phillyrin, M: 11-methyl-forsythide, and N forsythide. Red and blue color denote positive and negative correlations.

Based on the correlations shown in Figs 2 and 3, the *Forsythia* related metabolites were divided into two groups. Forsythiaside C, forsythenside B, forsythiaside J, forsythialan B, phillygenin, and phillyrin were all negatively or positively correlated with the other metabolites. However, the correlations between forsythoside B, rengyoside B, rengyoside A, forsythiaside A, isoforsythoside A, 11-methyl-forsythide, and forsythide and the other metabolites were inconsistent.

The *Forsythia* related metabolites and their substance types did not present any correlations. Phenolic acids were distributed in both classes, whereas only monoterpenoids and lignans were present in two classes. The correlations between metabolites of the same substance type and *Forsythia* related metabolites were inconsistent.

When ranked by forsythiaside, heatmaps of correlation values of *Forsythia* related metabolites showed a similarity of correlations between metabolites in fruits and leaves (Fig 3). Correlation values between other metabolites and *Forsythia* related metabolites in different plant organs exhibited similar results. These findings indicated the reproducibility of the potential metabolic pathways and the differentiation of metabolites. The results suggested that the metabolic pathways of *Forsythia* related metabolites could be divided into different groups.

### Molecular structures and cluster analysis of *Forsythia* related metabolites

**Fig 4:**
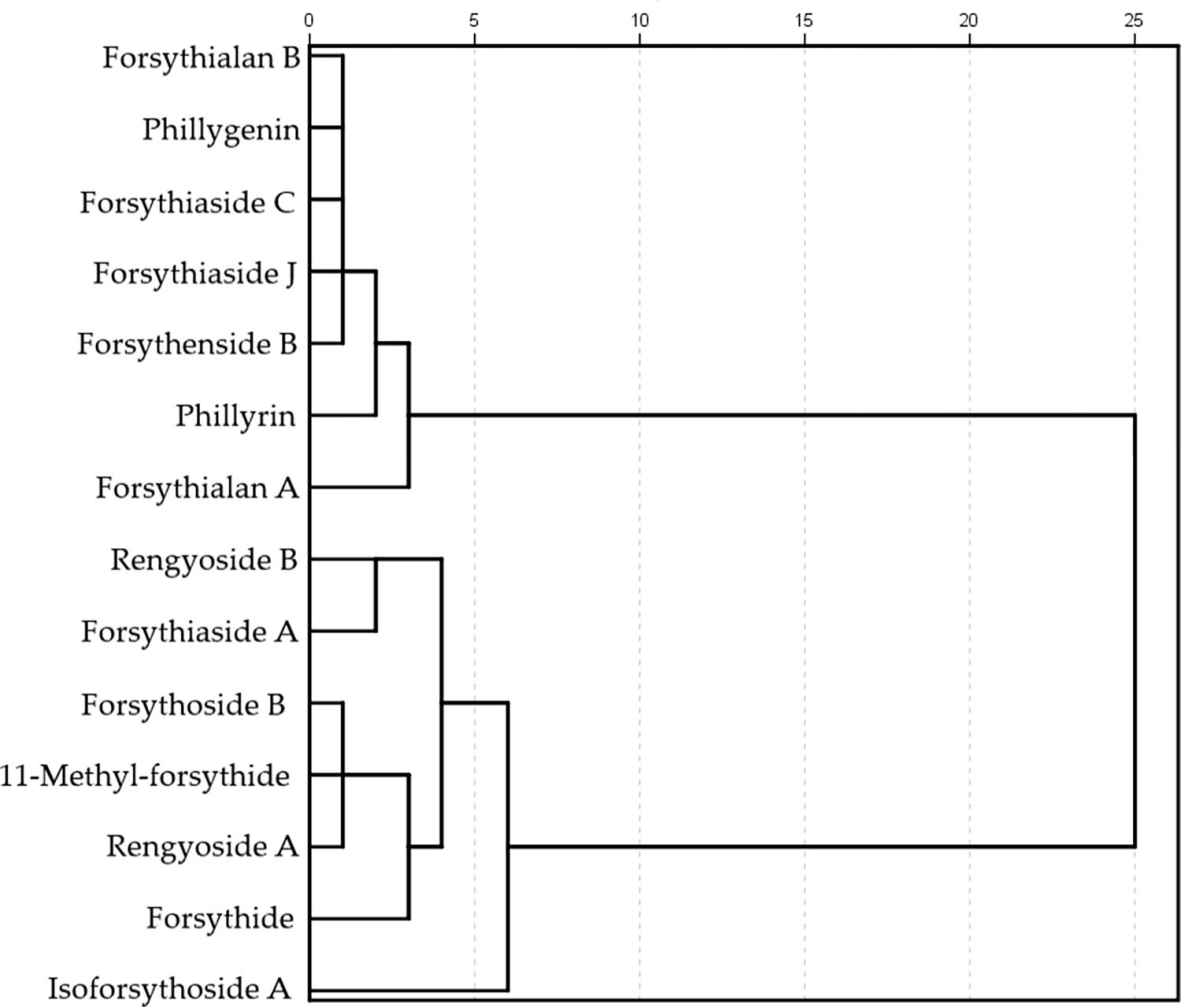
Results of cluster analysis of the *Forsythia* related metabolites in leaves and fruits in each harvesting period.

To further explore the relationships between the *Forsythia* related metabolites and other metabolites, a cluster analysis was performed (Fig 3). The *Forsythia* related metabolites could be divided into two classes, which were each further divided into two subclasses. *Forsythia* related metabolites in the first subclass of class 1 were forsythialan B, phillygenin, forsythiaside C, phillyrin, forsythiaside J, and forsythenside B. Only forsythialan A was clustered in the second class. The *Forsythia* related metabolites in the first subclass of the class 2 included rengyoside B, forsythiaside A, forsythoside B, 11-methyl-forsythide, rengyoside A, and forsythide. Only isoforsythoside A was clustered in the second subclass. The *Forsythia* related metabolites in classes 1 and 2 were lignans, phenolic acids, monoterpenoids, and phenolic acids. (Table 3, Fig 4).

**Table 3:** Classification of the *Forsythia* related metabolites.

Metabolite	Classification
Forsythiaside C	Phenolic acids
Forsythoside B	Phenolic acids
Rengyoside B	Phenolic acids
Rengyoside A	Phenolic acids
Forsythenside B	Phenolic acids
Forsythiaside J	Phenolic acids
Forsythiaside A	Phenolic acids
Isoforsythoside A	Phenolic acids
Forsythialan B	Lignans
Forsythialan A	Lignans
Phillygenin	Lignans
Phillyrin	Lignans
11-Methyl-forsythide	Monoterpenoids
Forsythide	Monoterpenoids

**Fig 5:**
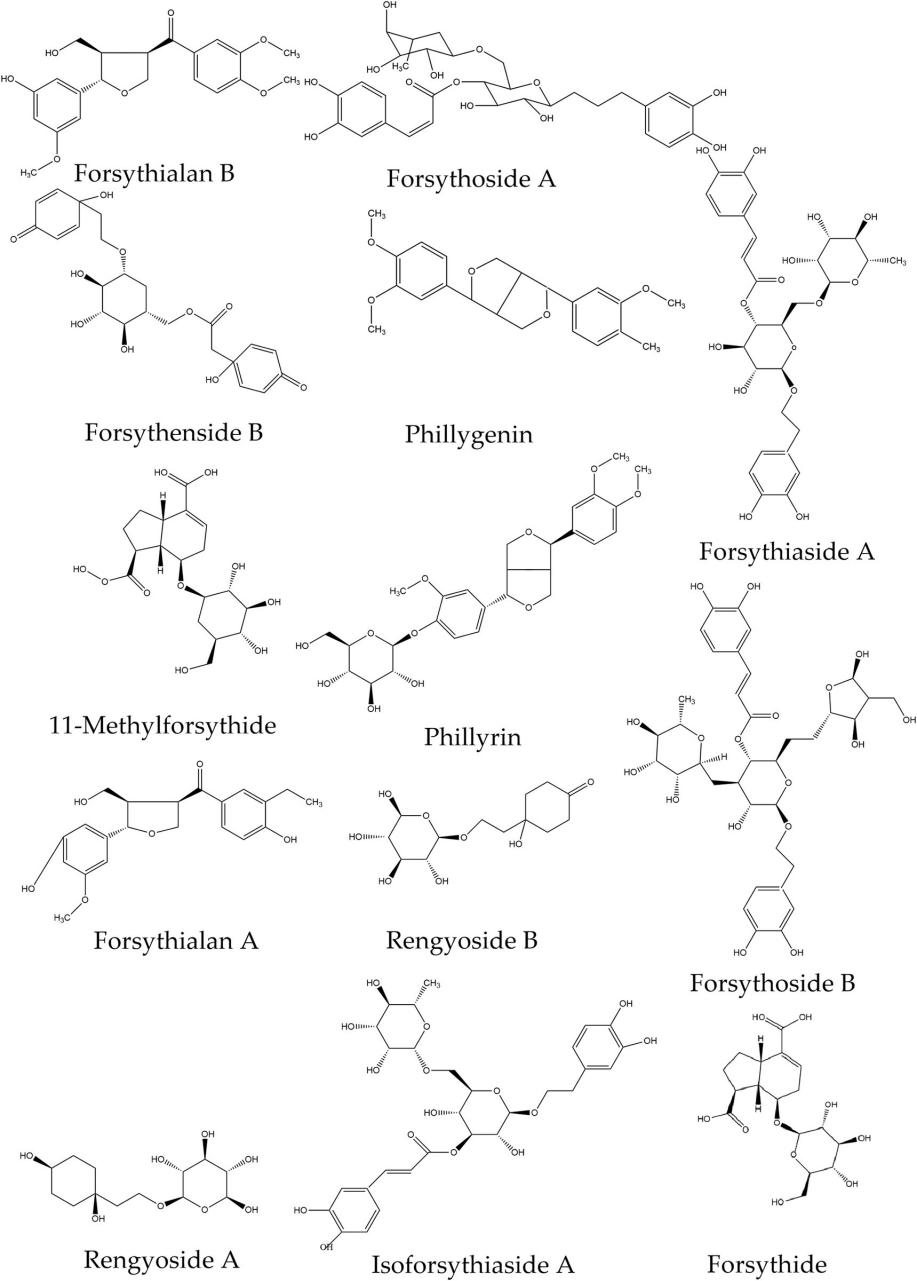
Molecular structures of *Forsythia* related metabolites.

The *Forsythia* related metabolites in the first class displayed two structures: 5-(4-(3,4-dimethoxyphenyl) tetrahydro-1H,3H-furo[3,4-c]furan-1-yl)-2-methoxyphenol (phillygenin) and (2R,3R,4S,5S,6S)-tetrahydro-2H-pyran-2,3,4,5,6-pentaol (Fig 5). The differentiation of phillyrin and phillygenin was caused by the insertion of (2R,3R,4S,5S,6S)-tetrahydro-2H-pyran-2,3,4,5,6-pentaol. The insertion may also occur during the biosynthesis processes of forsythialan A and forsythenside B. Forsythialan A, rengyoside B, phillygenin, and forsythialan B have similar molecular structures, and their differentiations may be due to hydroxylation. Forsythialan A and forsythenside B both have a hydroxyl group inserted, whereas forsythialan A also undergoes hydrogenation.

In the second subclass of *Forsythia* related metabolites, isoforsythoside A, forsythoside B, rengyoside A, forsythiaside A, rengyoside B, forsythide and 11-methyl-forsythide all displayed the same structure as (2R,3R,4S,5S,6S)-tetrahydro-2H-pyran-2,3,4,5,6-pentaol (Figs 4 and 5). Although 11-methyl-forsythide and forsythoside B were in the same cluster, but 11-methyl-forsythide was more likely to be a derivative of forsythide. In addition, methylation was observed in the molecular structures of 11-methyl-forsythide and forsythoside B. Methylations also occurred in the cluster of rengyoside B and forsythiaside A as well. Although forsythiaside A and isoforsythoside A have the same groups, cluster analysis revealed a distant relationship. However, isoforsythoside A also undergoes methylation.

### Screening of compounds significantly correlated with *Forsythia* related metabolites

A total of 33 compounds displayed significant correlations with nine *Forsythia* related metabolites in leaves and fruits (S5 Table). Nine metabolites had significant or highly significant positive correlations with forsythiaside C in the fruits and leaves during the fruit development process. Guanosine, cynarin, D-pinitol, (5S,8R,9Z,12Z)-5,8-dihydroxyoctadeca-9,12-dienoate, hispidulin, glucose-1-phosphate, 9-hydroxy-12-oxo-15(Z)-octadecenoic acid, D-glucose 6-phosphate, and (S)-2-phenyloxirane were all positively correlated with forsythiaside C. The metabolites were classified as nucleotides and their derivatives, phenolic acids, lipids, flavonoids, and others. Galactinol was positively correlated with forsythoside B in both fruits and leaves. Both of the metabolites were classified as phenolic acids. Rengyoside B was significantly or highly significantly positively correlated with hesperetin-6-C-glucoside-7-O-glucoside and D-panose. D- Galactaric acid was significantly negatively correlated with forsythenside B in fruits and had a highly significantly negative correlation in leaves. 2-α-Linolenoyl-glycerol-1,3-di-O-glucoside was positively correlated with forsythiaside J in both fruits and leaves. LysoPC 18:3 and 2-α-linolenoyl-glycerol-1,3-di-O-glucoside, which are both lipids, were negatively correlated with forsythiaside J, and 2-α-linolenoyl-glycerol-1,3-di-O-glucoside had a highly significant negative correlation (correlation coefficient = -0.999) in leaves. The *Forsythia suspensa* quality control compound forsythiaside A was positively correlated with uracil in both plant organs. The recently identified compound forsythialan B, was positively correlated with Z-6,7-epoxyligustilide, and esculetin, whereas it was negatively correlated with L-valine. Phillygenin and forsythialan B were classified as lignans and had similar patterns of correlation with the other metabolites. L-Valine had very similar correlations with phillygenin and forsythialan B. Forsythialan B and Z-6,7-epoxyligustilide were both positively correlated with phillygenin. Phillyrin was significantly negatively or positively correlated with 10 metabolites. Only chestnutlignansoide and calycosin were positively correlated with phillyrin, β-amyrenone, D-glucurono-6,3-lactone, sedoheptulose, citric acid, D-galacturonic acid, uracil and δ-amyrenone, and euscaphic acid had significant or extremely significant negative correlations with phillyrin.

## Discussion

In this study, *Forsythia* related metabolites in leaves and fruits during different fruit development stages were analyzed. The goal was to clarify the optimal harvesting time necessary to achieve a high content of medicinal compounds, and to establish the plant organs with the highest metabolite contents.

*Forsythia suspensa* has multiple medical applications because of its complex mixture of metabolites in leaves and fruits [25–27]. Significant differences in plant metabolic processes among plant organs have been described. Studies on *F*. *suspensa* have challenged the belief that the fruit is the main source of medicinal compounds, with leaves reportedly being a better source [26]. In the present study, the levels of phillyrin, forsythiaside A, isoforsythoside A, and forsythide in leaves were higher than those in fruits, consistent with the results of some previous studies, whereas the levels of forsythenside B, forsythoside B, forsythialan A, forsythialan B, and forsythiaside J were higher in fruits than in leaves. These compounds derived from leaves may be less suitable for medical use. The levels of the *Forsythia* related metabolites differed among development stages and plant organs, which is an important consideration for harvesting.

Studies on *Forsythia* related compounds have indicated that their main effects are antibacterial, antifungal, antiviral, antioxidant, anti-inflammatory, antitumor, and hypolipidemic activities [2, 21, 27–30]. Different compounds have different medicinal effects.

Isoforsythoside A and forsythiaside A have been reported to have inhibitory effects on *Staphylococcus aureus* and other pathogenic bacteria [31]. One study indicated that these molecules also have potent activities against fungi and influenza A virus [32]. *Forsyhia suspensa* metabolites also have antiseptic properties. Forsythin has been reported to exhibits anti-oxidation activity, lowers blood lipid levels and inhibits the oxidation of low-density lipoprotein [33]. Another study demonstrated that phillyrin can enhance the scavenging of 2,2-diphenyl-1-picrylhydrazyl (DPPH) and 3-ethylbenzothiazoline-6-sulfonic acid (ABTS) free radicals [34]. The present study confirmed differences in the content of *Forsythia* related metabolites between leaves and fruits. Different therapeutic effects can be expected between compounds derived from the leaves and fruits of *F*. *suspensa*. The plant organs from which compounds are extracted and the harvesting stage should be considered to ensure that the desired medicinal effects are achieved. We suggest that the leaves of *Forsythia suspensa* harvested at an intermediate stage, would likely provide the greatest medicinal effects because of their high levels of forsythiaside A, isoforsythoside A, phillygenin, and phillyrin.

Significant progress has been made in determining the components of medicinal herbs with medical applications, and the biosynthetic pathways of podophyllotoxin and colchicine have been identified [35, 36]. Studies on colchicine and podophyllotoxin have indicated that a combined metabolomic and transcriptomic analysis approach is suitable for studying biosynthetic pathways. Metabolomics is the major tool for the identification and screening of target substances, and determination of their levels in different plants. However, the biosynthetic pathways in *F*. *suspensa* are still not fully understood. Although previous studies have involved only enzyme assays, metabolic research offers much promise as a means of understanding the basis of biosynthetic pathways. A previous transcriptome study of the biosynthesis pathways of lignans performed in *Forsythia koreana* revealed that pinoresinol-lariciresinol reductase (PLR) and matairesinol *O*-methyltransferase (MOMT) from this plant catalyzed the biosynthesis of lariciresinol from pinoresinol and biosynthesis of larctigenin from matairesinol [37]. Phillygenin, forsythialan A and forsythialan B were all classified into one cluster by analyzing their correlations. PLR and MOMT regulate the transformations of pinoresinol to lariciresinol and of matairesinol to arctigenin, which are similar to the transformation of phillgenin to forsythialan A and from forsythialan A to forsythialan B. Thus, PLR and MOMT may be involved in the biosynthesis of distinctive metabolites of *Forsythia*. Because PLR and MOMT of *F*. *koreana* participate in the biosynthesis processes of forsythialan A and forsythialan B, we assumed that forsythialan A and forsythialan B are the downstream metabolites of phillygenin. We suggest that the medicinal functions of phillygenin, forsythialan A and forsythialan B should be further studied to verify the functions of PLR and MOMT in *Forsythia suspensa*. Ying et al. [38] reported the different catalytic efficiencies and specificities of pinoresinol-lariciresinol reductases and pinoresinol reductases (PrRs) in *Arabidopsis thaliana* and *Isatis indigotica* by analyzing and comparing the crystal structures of PLRs and pinoresinol reductases. The findings indicated that the different crystal structures affect the catalytic efficiencies and selectivities. PLR1 from *I*. *indigotica* and *A*. *thaliana* reduce pinoresinol and lariciresinol with different catalytic efficiencies, wherease pinoresinol reductase 2 from *A*. *thaliana* only pinoresinol to produce lariciresinol. Thus, the crystal structures of PLRs in different species might be different, which results in different metabolism of lignans and the production of differing amounts of a variety of active substances [38, 39]. We assume that the PLRs in *Forsythia* may from *A*. *thaliana* reduces have important roles in the biosynthesis of *Forsythia* specific metabolites. The metabolic functions of PLRs in *Forsythia* need to be further studied. Our study revealed positive correlations between forsythoside B and 11-methyl-forsythide and forsythialan B and phillygenin, in both leaves and fruits. Thus, there might be commonalities among these metabolites in terms of their biosynthetic pathways and precursors. Cluster analysis of the metabolites indicated that the two major classes identified may be subject to similar biosynthetic processes. Phenolic acids were present in both classes, whereas lignans and terpenoids were present in two different subclasses respectively. Correlation analysis revealed both positive and negative correlations among metabolites, but only a few of these significant or highly significant. Consistent correlations among *Forsythia* related metabolites within a single class indicated that their biosynthetic pathways may have similar processes in upstream metabolism. The chemical structures of the metabolites in each subclass further indicated the differentiation of the biosynthetic pathways. Although the chemical structures of many metabolites have already been identified, their biosynthetic pathways remain poorly understood. Phenolic acids presented in both classes, and their biosynthetic processes may play important roles in their upstream metabolism. Terpenoids and lignans were clustered into different subclasses separately. The biosynthetic pathways of the lignans and terpenoids may have significant effects on metabolites differentiation. The biosynthetic pathways of the *Forsythia* related metabolites were significantly different, and there might be different upstream biosynthetic processes in the two subclasses. Differences in synthesis and differentiation of downstream products may underlie the different metabolite compositions of the two classes. In the present study, forsythialan B and phillygenin, 11-methyl-forsythide and forsythoside B showed negative or positive correlations, respectively. The cluster analysis also showed that forsythialan B and phillygenin 11-methyl-forsythide and forsythoside B may have similar processes in their biosynthesis pathways, which also confirmed the validity of the correlation and cluster analysis. Forsythialan B, phillygenin, forsythiaside C, forsythiaside J, forsythiaside B, phillyrin and forsthialan A were clustered in one subclass. However, forsythialan B, phillygenin, phillyrin and forsythialan A were identified as lignans. Although the other metabolites were phenolic acids, and phenolic acids present in both subclasses. Thus, we suggest that the studies on metabolites of these lignans should focus on the transformations from phenolic acid to lignans and the differentiations of their chemical groups, such as methoxy and hydroxyl. Forsythide and 11-methylforsythide were monoterpenoids, and clustered into one subclass. Their chemical structures and cluster results showed similarities in biosynthesis processes. Enzymes that catalyze the insertion of the ether bond may facilite the biosynthesis of 11-methylforsythide. In addition, phillygenin was identified to play an important role in the biosynthesis pathways of the *Forsythia* related metabolites, and hydroxylation-related genes and enzymes may be involved in the processes of forsythialan A and forsythialan B. Tetrahydro-1H,3H-furo[3,4-c]furan was a common group of the *Forsythia* related metabolites in the first subclass. *Forsythia* related metabolites of the second subclass did not have similar furan groups. Thus, we assume that the biosynthesis and insertion of tetrahydro-1H,3H-furo[3,4-c]furan may occur upstream. (2R,3R,4s,5S,6S)-tetrahydro-2H-pyran-2,3,4,5,6-pentaol, was found in most of the *Forsythia* related metabolites as a common group. Its biosynthesis may occur upstream, as there was no indication of its formation in the differentiation process of the *Forsythia* related metabolites. As only ether bond of the common group, (2R,3R,4s,5S,6S)-tetrahydro-2H-pyran-2,3,4,5,6-pentaol, of 11-methylforsythide was replaced with a methylene. We assume that compared with other *Forsythia* related metabolites, 11-methylforsythide was a downstream metabolite. As phillyrin had both of two common groups, its biosynthesis may be a cross procedure among the biosyntheses of the two subclasses of *Forsythia* related metabolites. Cluster analysis and molecular structures indicated that there are the potential biosynthesis orders of the *Forsythia* related metabolites.

In this study, we analyzed the *Forsythia* related constituents in leaves and fruits at different harvesting stages. The findings support the suggestion that harvesting should be timed with the metabolites contents. The stability and differences in metabolism of forsythiaside A and phillyrin levels during fruit development could facilitate the identification of the two classes of *Forsythia* related metabolites reported in this study. This confirms the rationality of the quality evaluation method for *F*. *suspensa* in the Chinese Pharmacopeia. This study provides a theoretical basis for further study of the *Forsythia* related metabolites in *F*. *suspensa*, including in terms of biosynthesis and metabolic pathways.

## Conclusions

Different metabolites levels and proportions of metabolites may influence the medicinal properties of *F*.*suspensa*. We found that the metabolites levels in both fruits and leaves varied during different developmental periods. Thus, the harvest timing and plant organs should be carfully considered. Correlation and cluster analyses indicated two major types of potential biosynthesis pathways of the *Forsythia* related metabolites in *F*.*suspensa*. (2R,3R,4s,5S,6S)-Tetrahydro-2H-pyran-2,3,4,5,6-pentaol and phillygenin are two typical molecular structures of *Forsythia* related metabolites. The related enzymes and genes involved in the insertion of (2R,3R,4s,5S,6S)-tetrahydro-2H-pyran-2,3,4,5,6-pentaol may play important roles in the formation of both types of *Forsythia* related metabolites. Related hydroxylation enzymes and hydrogenases may be involved in the differentiation of metabolites into the first type.

## Supporting information

S1 Fig Total ion chromatogram of quality control samples in positive ion mode. (DOCX)

S2 Fig Total ion chromatogram of quality control samples in negative ion mode. (DOCX)

S3 Fig Multi-peak detection plot of metabolites acquired in positive ion multiplereaction monitoring mode. (DOCX)

S4 Fig Multi-peak detection plot of metabolites acquired in negative ion multiple reaction monitoring mode. (DOCX)

S5 Fig T1 of fruits_XIC_detection_of_multimodal_maps-N. (PDF)

S6 Fig T1 of fruits_XIC_detection_of_multimodal_maps-P. (PDF)

S7 Fig T1 of fruits_QC_MS_TIC-N. (PDF)

S8 Fig T1 of fruits_QC_MS_TIC-P. (PDF)

S9 Fig T1 of leaves_XIC_detection_of_multimodal_maps-P. (PDF)

S10 Fig T1 of leaves_XIC_detection_of_multimodal_maps-N. (PDF)

S11 Fig T1 of leaves_QC_MS_TIC-N. (PDF)

S12 Fig T1 of leaves_QC_MS_TIC-P. (PDF)

S13 Fig T2 of fuits_XIC_detection_of_multimodal_maps-N. (PDF)

S14 Fig T2 of fuits_XIC_detection_of_multimodal_maps-P. (PDF)

S15 Fig T2 of fuits_QC_MS_TIC-N. (PDF)

S16 Fig T2 of fuits_QC_MS_TIC-P. (PDF)

S17 Fig T2 of leaves_XIC_detection_of_multimodal_maps-N. (PDF)

S18 Fig T2 of leaves_XIC_detection_of_multimodal_maps-P. (PDF)

S19 Fig T2 of leaves_QC_MS_TIC-N. (PDF)

S20 Fig T2 of leaves_QC_MS_TIC-P. (PDF)

S21 Fig T3 of fruits_XIC_detection_of_multimodal_maps-N. (PDF)

S22 Fig T3 of fruits_XIC_detection_of_multimodal_maps-P. (PDF)

S23 Fig T3 of fruits_QC_MS_TIC-N. (PDF)

S24 Fig T3 of fruits_QC_MS_TIC-P. (PDF)

S25 Fig T3 of leaves_XIC_detection_of_multimodal_maps-N. (PDF)

S26 Fig T3 of leaves_XIC_detection_of_multimodal_maps-P. (PDF)

S27 Fig T3 of leaves_QC_MS_TIC-N. (PDF)

S28 Fig T3 of leaves_QC_MS_TIC-P. (PDF)

S29 Fig T4 of leaves_XIC_detection_of_multimodal_maps-N. (PDF)

S30 Fig T4 of leaves_XIC_detection_of_multimodal_maps-P. (PDF)

S31 Fig T4 of leaves_QC_MS_TIC-N. (PDF)

S32 Fig T4 of leaves_QC_MS_TIC-P. (PDF)

S33 Fig T4 of fruits_XIC_detection_of_multimodal_maps-N. (PDF)

S34 Fig T4 of fruits_XIC_detection_of_multimodal_maps-P. (PDF)

S35 Fig T4 of fruits_QC_MS_TIC-N. (PDF)

S36 Fig T4 of fruits_QC_MS_TIC-P. (PDF)

S1 Table Ion pair information. (DOCX)

S2 Table Correlations of metabolites in *Forsythia suspensa* fruits. (XLSX)

S3 Table Correlations of metabolites in *Forsythia suspensa* leaves. (XLSX)

S4 Table Screened metabolites of accordant expressed in fruits and leave of *Forsythia suspensa*. (XLSX)

S5 Table Analysis of compounds significantly correlated with *Forsythia suspensa* related metabolites. (XLSX)
