# Supplementary figures and images for: Metabolome analysis of genus Forsythia related constituents in Forsythia suspensa leaves and fruits using UPLC-ESI-QQQ-MS/MS technique
Source: PLoS One. 2022 Jun 28;17(6):e0269915. doi: 10.1371/journal.pone.0269915 (PMC9239459; doi:10.1371/journal.pone.0269915)

**
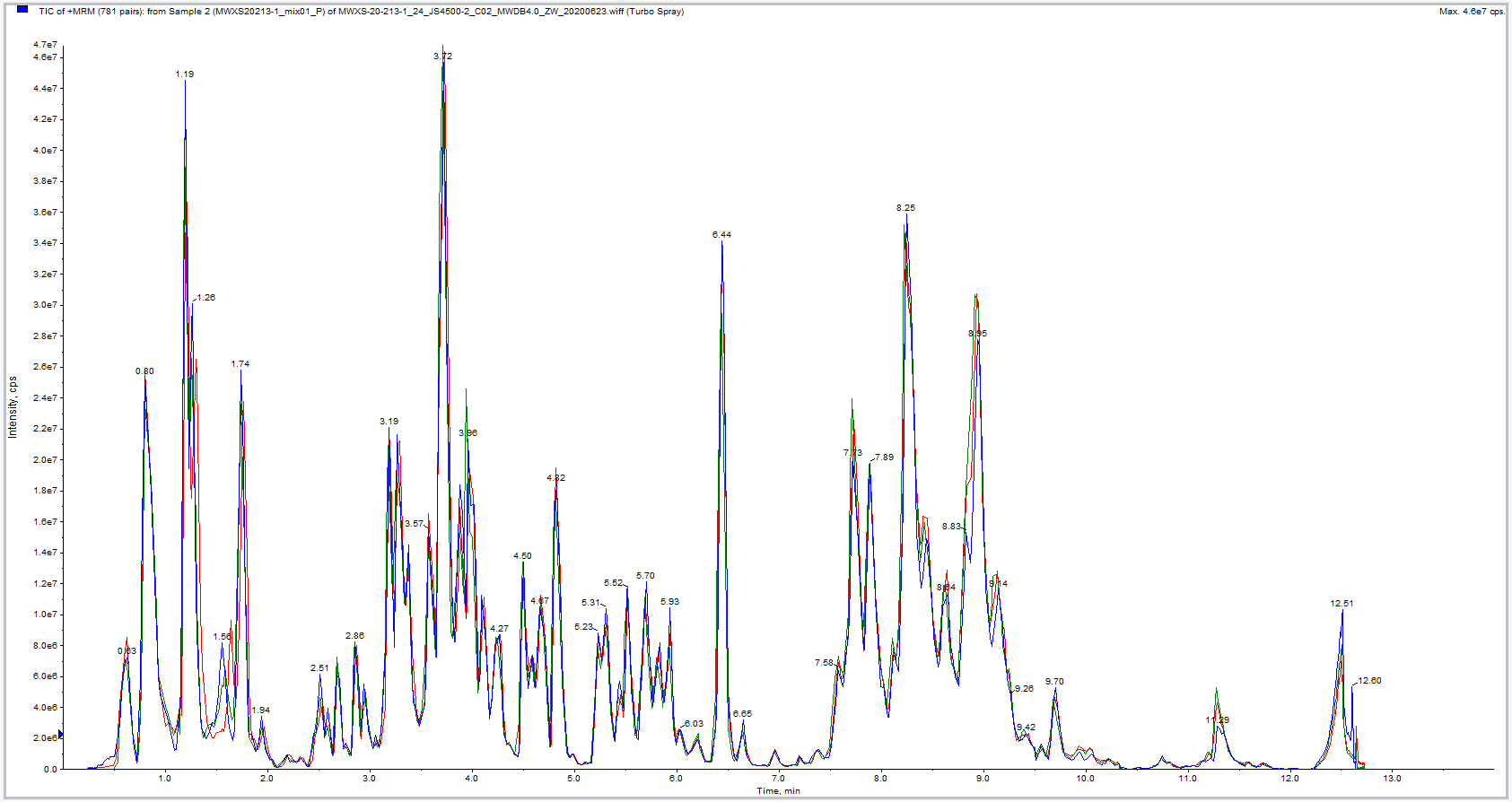
**

**S1 Fig. Total ion chromatogram of quality control samples in positive ion mode**

Supplement: S1 Fig — (DOCX) [file pone.0269915.s001.docx]

**
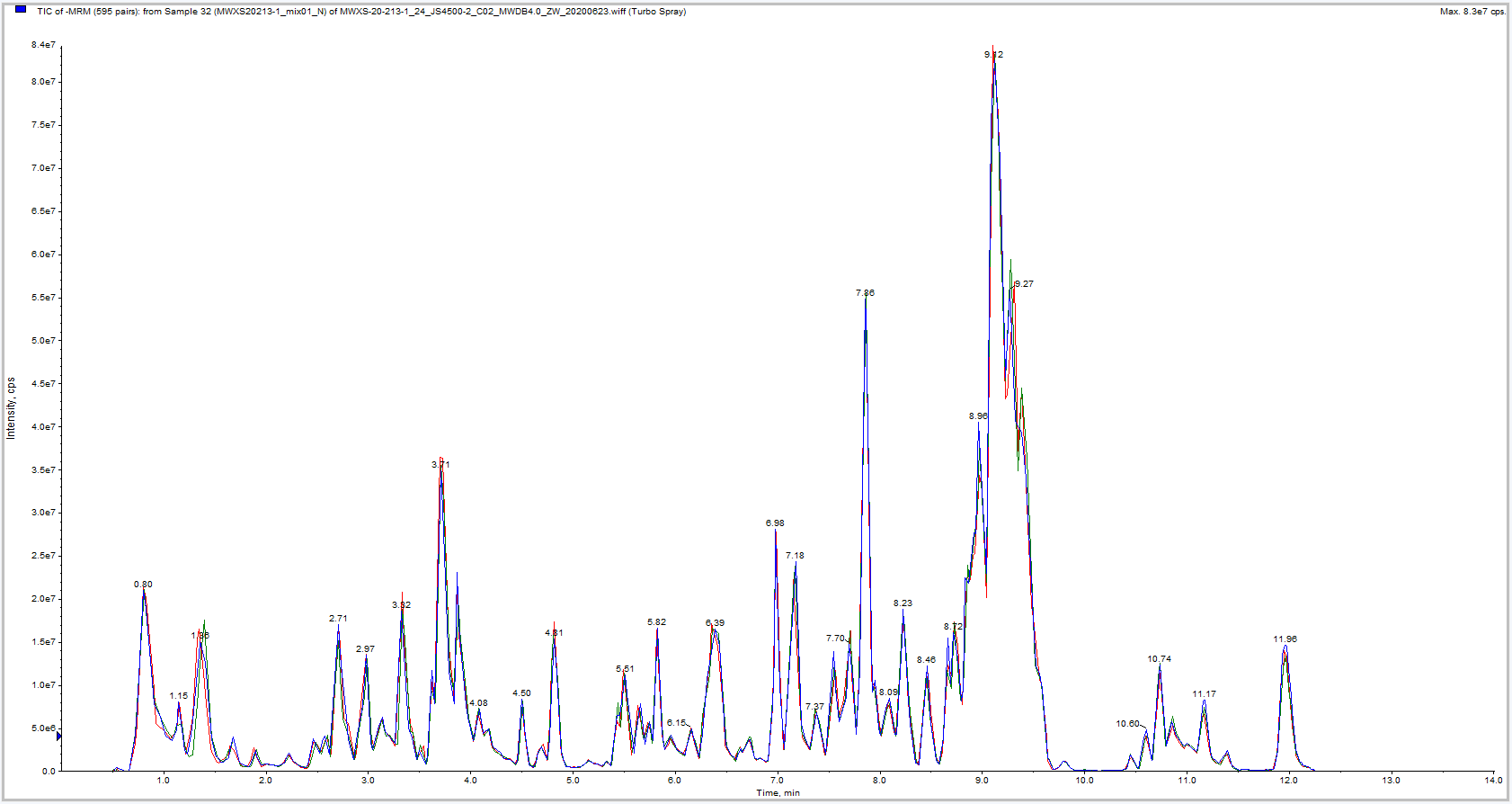
**

**S2 Fig. Total ion chromatogram of quality control samples in negative ion mode**

Supplement: S2 Fig — (DOCX) [file pone.0269915.s002.docx]

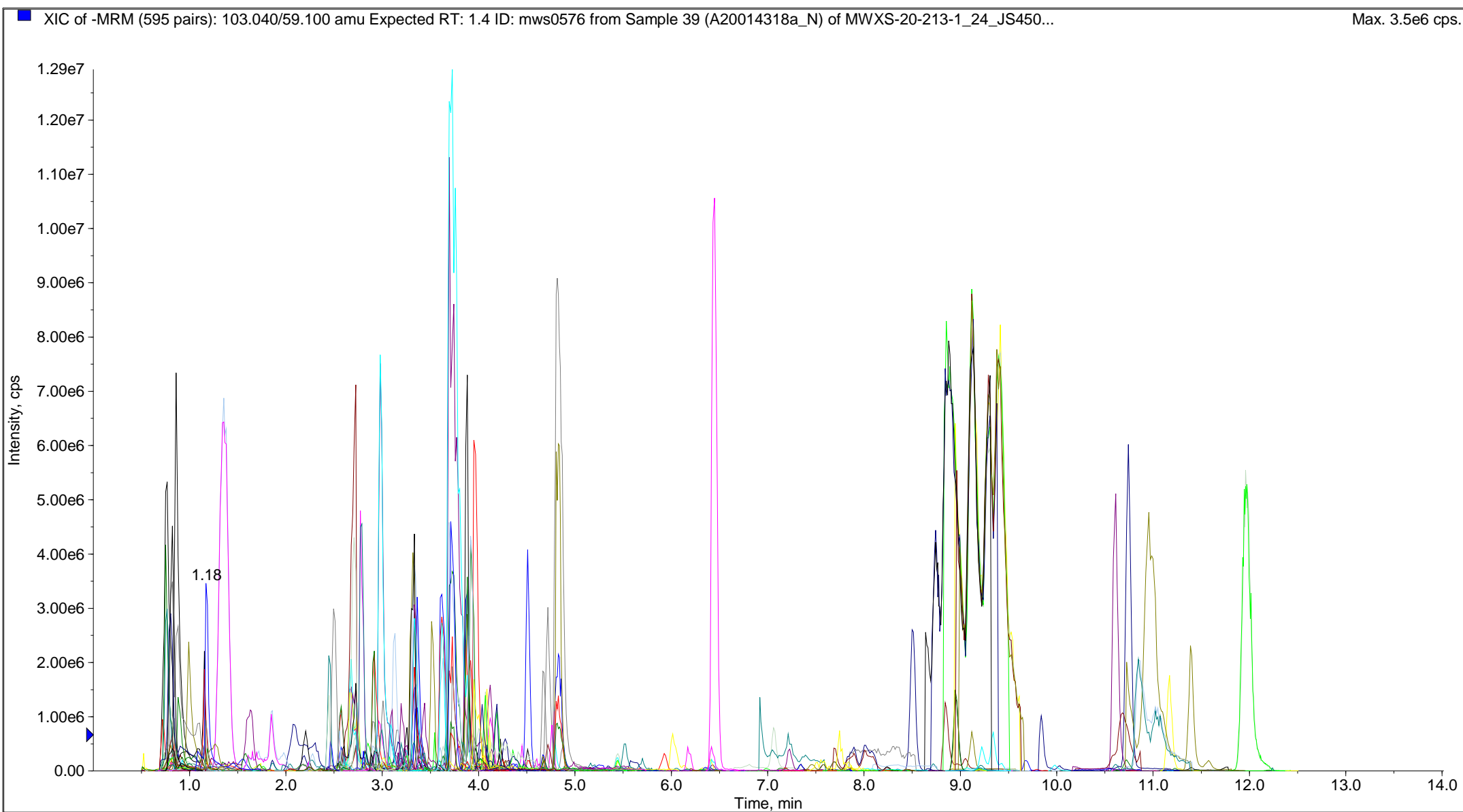

S17 Fig. T2 of leaves\_XIC\_detection\_of\_multimodal\_maps-N

Supplement: S17 Fig — (PDF) [file pone.0269915.s017.pdf]

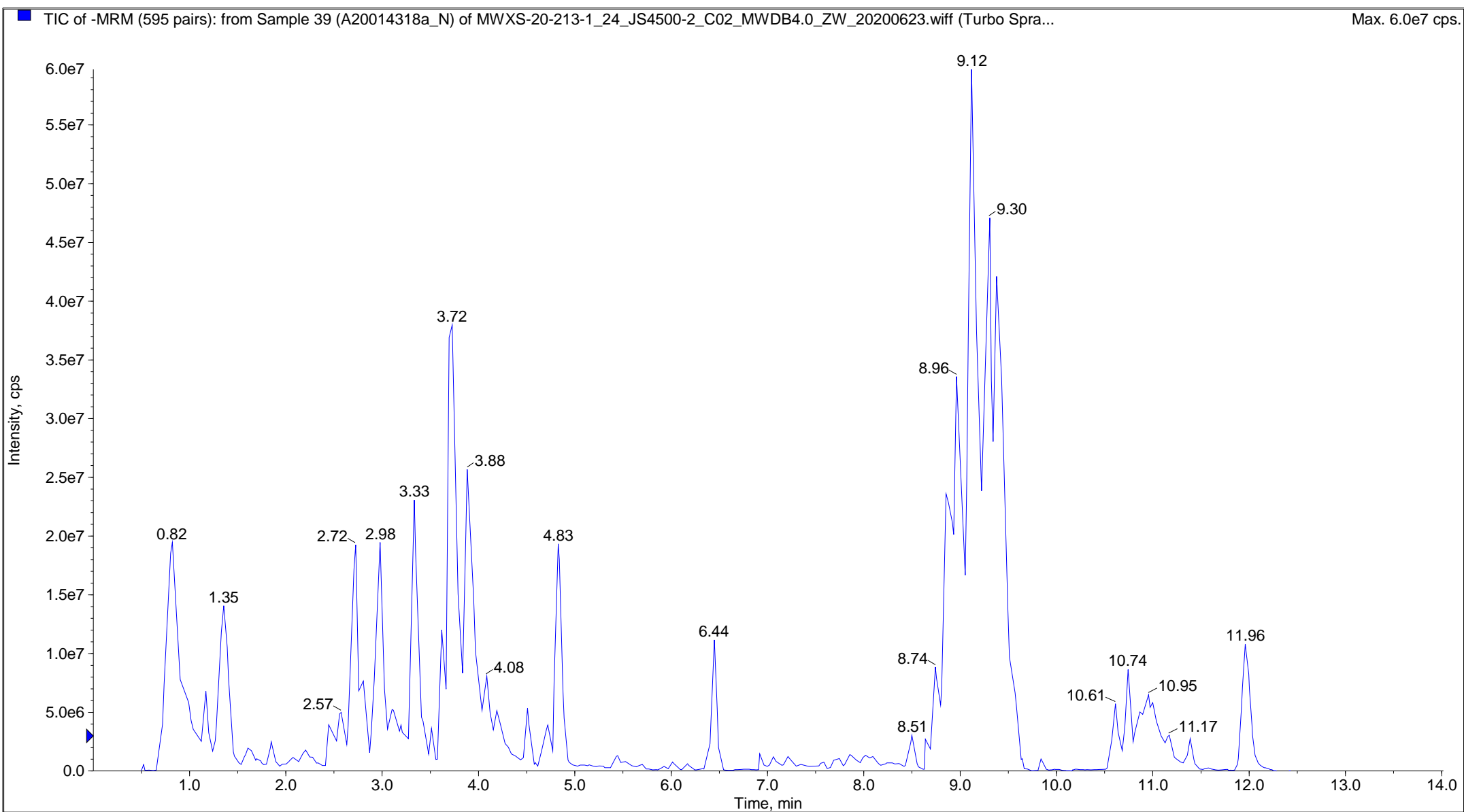

S19 Fig. T2 of leaves\_QC\_MS\_TIC-N

Supplement: S19 Fig — (PDF) [file pone.0269915.s019.pdf]
