# Supplementary material for: Metabolome analysis of genus Forsythia related constituents in Forsythia suspensa leaves and fruits using UPLC-ESI-QQQ-MS/MS technique
Source: PLoS One. 2022 Jun 28;17(6):e0269915. doi: 10.1371/journal.pone.0269915 (PMC9239459; doi:10.1371/journal.pone.0269915)
Supplement: S4 Fig — (DOCX) [file pone.0269915.s004.docx]

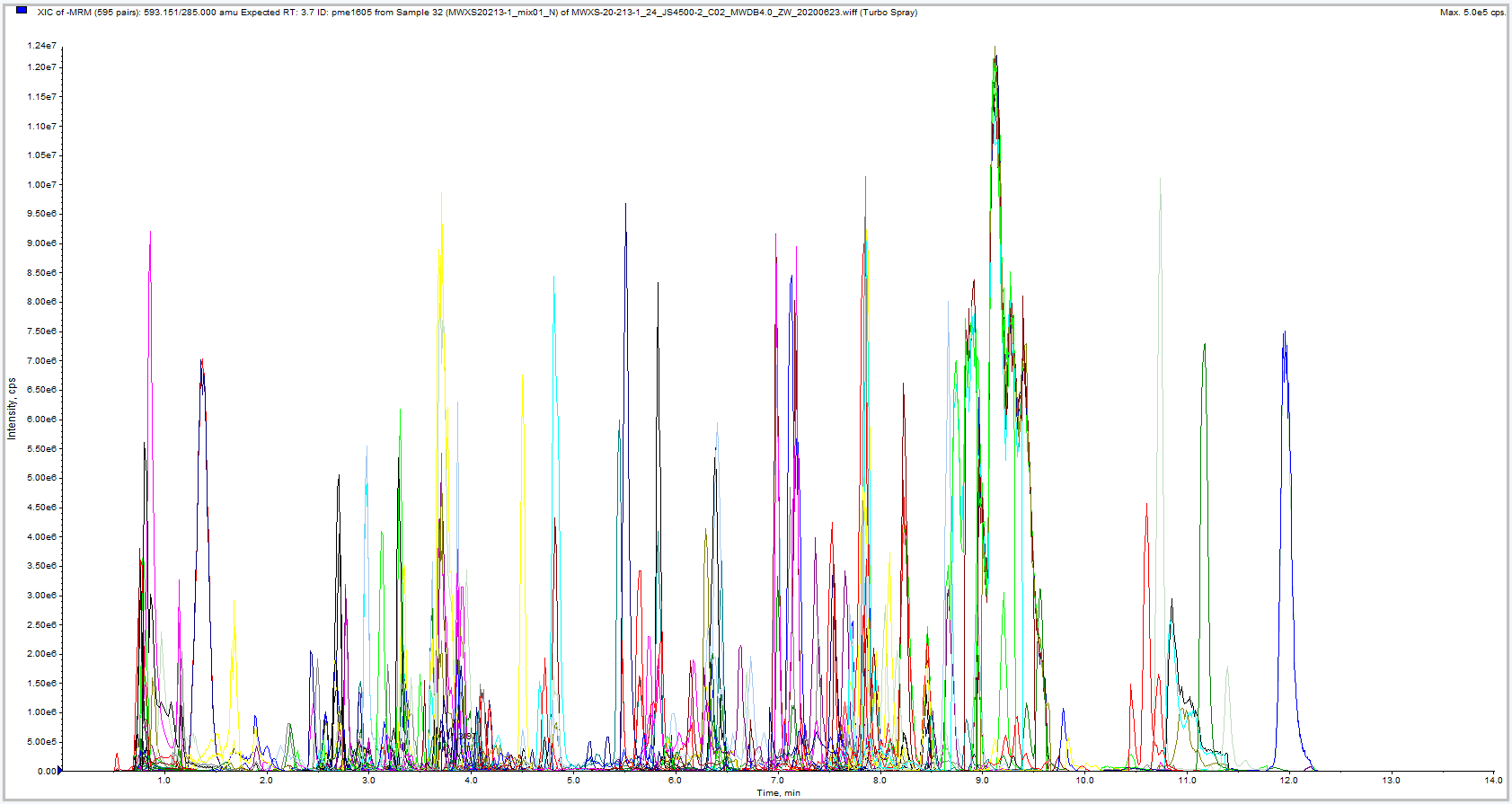


**S4 Fig. Multi-peak detection plot of metabolites acquired in negative ion multiple reaction monitoring mode**
