# Supplementary material for: Metabolome analysis of genus Forsythia related constituents in Forsythia suspensa leaves and fruits using UPLC-ESI-QQQ-MS/MS technique
Source: PLoS One. 2022 Jun 28;17(6):e0269915. doi: 10.1371/journal.pone.0269915 (PMC9239459; doi:10.1371/journal.pone.0269915)
Supplement: S26 Fig — (PDF) [file pone.0269915.s026.pdf]

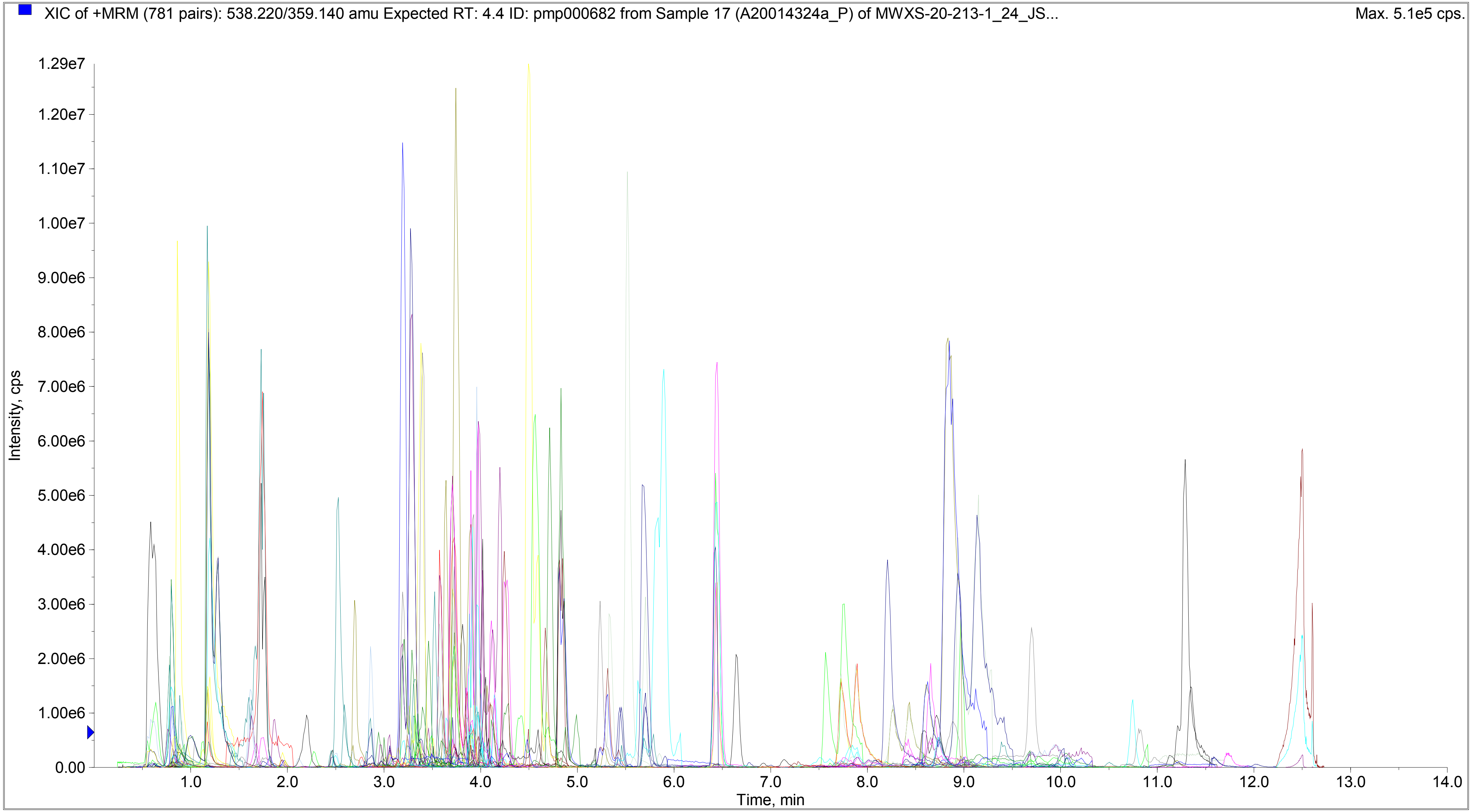

S26 Fig. T3 of leaves_XIC_detection_of_multimodal_maps-P
