# Supplementary material for: Metabolome analysis of genus Forsythia related constituents in Forsythia suspensa leaves and fruits using UPLC-ESI-QQQ-MS/MS technique
Source: PLoS One. 2022 Jun 28;17(6):e0269915. doi: 10.1371/journal.pone.0269915 (PMC9239459; doi:10.1371/journal.pone.0269915)
Supplement: S1 Table — (DOCX) [file pone.0269915.s037.docx]

**S1 Table.** **Ion pair information**

| **Compounds** | **Category** | **Molecular Weight (Da)** | **Ion mode** | **Ionization model** | **Q1**  **(Da)** | **Q3**  **(Da)** |
| --- | --- | --- | --- | --- | --- | --- |
| Forsythialan A | Lignans | 374.14 | positive | [M+H]+ | 375.1 | 151.3 |
| Forsythialan B | Lignans | 388.15 | positive | [M+H]+ | 389.2 | 165.1 |
| Forsythenside B | Phenolic | 484.2 | positive | [M+NH4]+ | 151.0 | 466.1 |
| Forsythiaside J | Phenolic | 611.2 | positive | [M+H]+ | 163.0 | 610.2 |
| Forsythiaside A | Phenolic | 623.2 | negative | [M-H]- | 161.0 | 624.2 |
| Isoforsythoside A* | Phenolic | 623.2 | negative | [M-H]- | 461.2 | 624.2 |
| Forsythiaside C* | Phenolic | 658.2 | Positive | [M+NH4]+ | 163.0 | 640.2 |
| Forsythoside B* | Phenolic | 755.2 | negative | [M-H]- | 593.2 | 756.7 |
| Forsythide | Monoterpenoids | 408.2 | positive | [M+NH4]+ | 211.1 | 390.1 |
| 11-methyl-forsythide* | Monoterpenoids | 422.3 | Positive | [M+NH4]+ | 243.1 | 404.1 |
| Phillygenin | Lignans | 373.2 | Positive | [M+H]+ | 337.1 | 372.2 |
| Phillyrin | Lignans | 533.2 | negative | [M-H]- | 371.0 | 534.2 |
| Rengyoside B | Phenolic | 321.2 | Positive | [M+H]+ | 141.1 | 320.2 |
| Rengyoside A | Phenolic | 323.2 | positive | [M+H]+ | 107.1 | 322.2 |

Q1: precursor ion; Q3: characteristic fragment ion
